# Specific cell surface labeling of GPCRs using split GFP

**DOI:** 10.1038/srep20568

**Published:** 2016-02-09

**Authors:** Wen-Xue Jiang, Xu Dong, Jing Jiang, Yu-Hong Yang, Ju Yang, Yun-Bi Lu, San-Hua Fang, Er-Qing Wei, Chun Tang, Wei-Ping Zhang

**Affiliations:** 1 Department of Pharmacology, Zhejiang University School of Medicine, Hangzhou, Zhejiang 310058, China; 2 CAS Key Laboratory of Magnetic Resonance in Biological Systems, State Key Laboratory of Magnetic Resonance and Atomic Molecular Physics, Wuhan Institute of Physics and Mathematics of the Chinese Academy of Sciences, Wuhan, Hubei Province 430071, China; 3 Core Facilities of Zhejiang University Institute of Neuroscience, Zhejiang University School of Medicine, Hangzhou, Zhejiang 310058, China

## Abstract

Specific cell surface labeling is essential for visualizing the internalization processes of G-protein coupled receptors (GPCRs) and for gaining mechanistic insight of GPCR functions. Here we present a rapid, specific, and versatile labeling scheme for GPCRs at living-cell membrane with the use of a split green fluorescent protein (GFP). Demonstrated with two GPCRs, GPR17 and CysLT_2_R, we show that two β-stands (β-stands 10 and 11) derived from a superfolder GFP (sfGFP) can be engineered to one of the three extracellular loop of a GPCR. The complementary fragment of sfGFP has nine β-strands (β-stands 1-9) that carries the mature fluorophore, and can be proteolytically derived from the full-length sfGFP. Separately the GFP fragments are non-fluorescent, but become fluorescent upon assembly, thus allowing specific labeling of the target proteins. The two GFP fragments rapidly assemble and the resulting complex is extremely tight under non-denaturing conditions, which allows real-time and quantitative assessment of the internalized GPCRs. We envision that this labeling scheme will be of great use for labeling other membrane proteins in various biological and pharmacological applications.

G-protein coupled receptors (GPCRs) constitute the main family of cell-surface receptors in mammalian cells^1^, and over 30% of the pharmaceutical targets are GPCRs^2^. When activated, a GPCR interacts with intracellular or transmembrane proteins, thereby triggering downstream signals. In addition, a GPCR can be internalized via a recycling or degradation pathway, thus dampening the signal^3^,^4^. The biology of when and how a GPCR is internalized is of great physiological, pathophysiological and pharmacological interest^5^. To visualize this cellular process in real-time, specific cell surface labeling is required.

To label a membrane protein, a variety of chemical, biochemical, and immunological labeling methods have been developed^6^. Genetically encoding of a fluorescent protein, such as green fluorescent protein (GFP) or mCherry, is also widely used for membrane protein labeling. For GPCRs, the GFP has been engineered to the N-terminus, the C-terminus, or to the third intracellular loop of a GPCR protein^5^,^7^. Nevertheless, because the GPCR termini and intracellular loops can be involved in ligand binding and signal transduction and because a GFP has a molecular weight of 27 kDa, engineering of the full-length GFP may inadvertently perturb GPCR function^8^. Further, as GFP-fused GPCRs can be visualized both at the cell membrane and within the cell, and therefore GPCR internalization cannot be adequately assessed using the standard GFP engineering approach.

A GFP comprises eleven β-stands arranged in a barrel structure, and the GFP fluorophore is protected inside the barrel from solvent exposure and fluorescence quenching. Regan and colleagues demonstrated that it was possible to split the GFP into two non-fluorescent fragments that could be subsequently reassembled with the help of a Leucine zipper^9^. The split GFP technique has been further developed through the engineering of what is known as the “superfolder GFP” (sfGFP), a modified version of GFP that confer improved stability^10^. Waldo and colleagues expressed the two fragments of sfGFP that contain β-strands 1-10 and β-stands 11, respectively. Neither fragment fluoresces by itself; but when mixed, the two fragments spontaneously assemble and become fluorescent^10^. Split GFP technique based on sfGFP has been used to evaluate protein-protein interactions^11^,^12^, to assess protein folding and solubility^13^,^14^, and also for the fluorescent labeling of intracellular proteins^14^,^15^,^16^. Yet, the split GFP technique has not been used for the labeling of GPCRs. Inspired by the split GFP design, we here present a novel split GFP scheme for rapid, specific, and versatile fluorescent labeling of a GPCR at the surface of living cells.

## Results

### Design of the specific cell-surface labeling scheme

**Figure 1:**
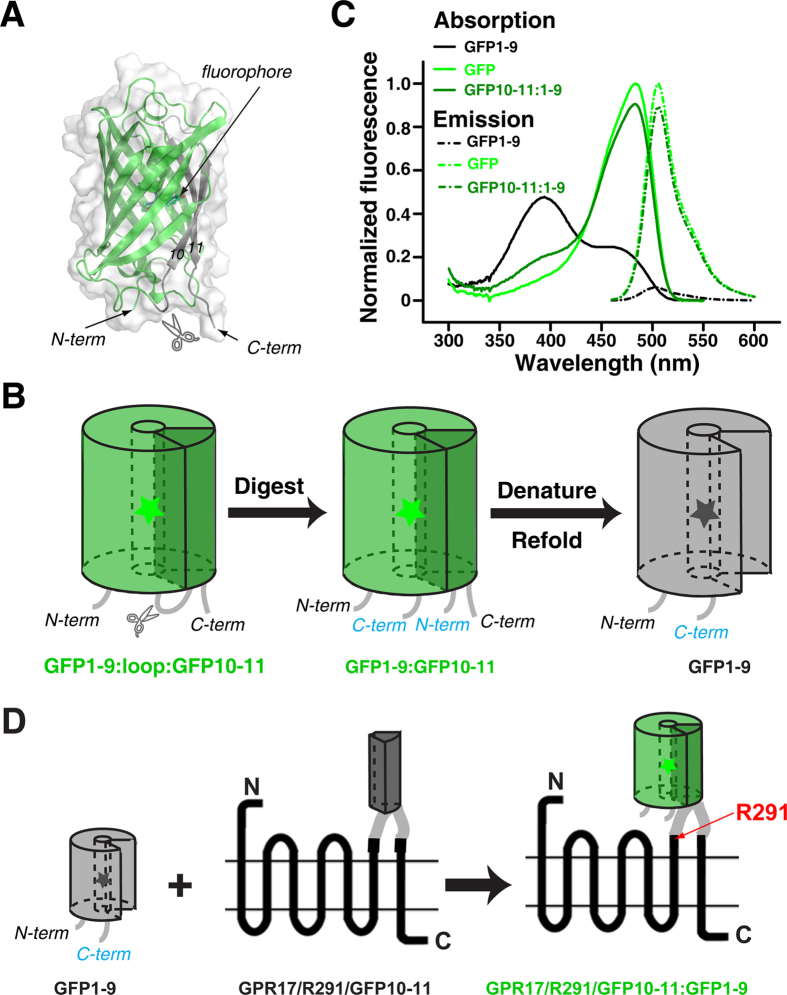
Illustration of the split GFP scheme for the cell surface labeling of a GPCR protein. (**A**) GFP structure, with GFP β-strands 1-9 (GFP1-9) colored in green and GFP β-strands 10-11 (GFP10-11) colored in gray. A trypsin cleavage site was introduced between the 9^th^ and 10^th^ β-strands, as indicated. (**B**) Schematic diagram for the preparation of GFP1-9. Following trypsin digestion, the two fragments were separated in the presence of 3 M guanidine hydrochloride. GFP1-9 was then purified and refolded. (**C**) Absorption and fluorescence emission spectra for wild type GFP, GFP1-9, and GFP10-11:GFP1-9 complex. (**D**) Schematic diagram for the assembly between GFP1-9 and GFP10-11 engineered to the third extracellular loop of GPR17, a GPCR protein.

We split the sfGFP protein into two fragments, the large one containing β-strands 1 to 9 (referred to as GFP1-9) and the small one containing β-strands 10 and 11 (referred to as GFP10-11). To obtain GFP1-9, we engineered a trypsin cleavage site between the β-strands 9 and 10 of sfGFP (Fig. 1A). Upon trypsin digestion, the two GFP fragments are tightly held together and could only be separated with the use of 3M guanidine hydrochloride. After denaturation, GFP1-9 could be purified and refolded (Fig. 1B). Proteolytically derived from the full-length sfGFP, the GFP1-9 fragment harbors an entire mature fluorophore. Nevertheless, the fluorophore in GFP1-9 is solvent exposed, and therefore GFP1-9 is non-fluorescent by itself (Fig. 1B,C). When GFP1-9 is reassembled with GFP10-11 and the β-barrel structure is re-established, the GFP fluorophore is again shielded from solvent and immediately becomes fluorescent. The absorption and emission spectra of reassembled GFP are similar to those of the full length GFP (Fig. 1C).

GFP10-11 can be genetically engineered to a loop of a target protein as a small protein tag. The GFP10-11 contains two β-strands, which can protrude out away from the target protein and cause minimal structural and functional perturbation. In addition, the GFP10-11 tag can readily interact with GFP1-9 administered in solution with little steric hindrance (Fig. 1D). Furthermore, as a GPCR has three extracellular loops, the GFP10-11 tag can be optimally engineered to any one of these loops.

### GFP1-9 complements GFP10-11 engineered to a loop of a soluble protein

**Figure 2:**
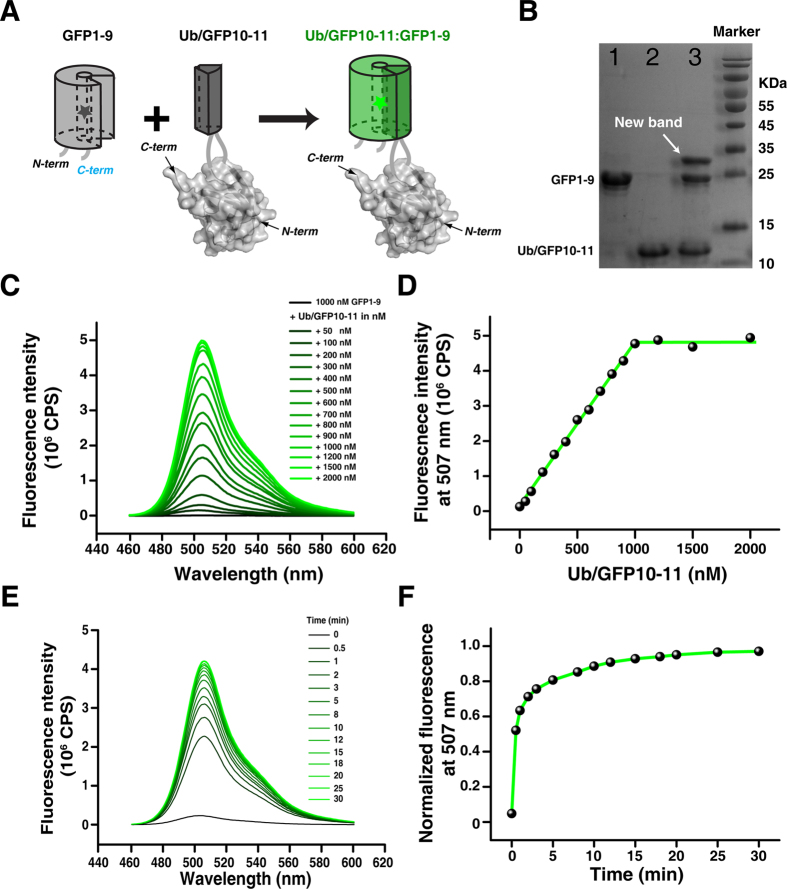
The assembly between GFP1-9 and GFP10-11 in solution. (**A**) Schematic diagram showing the assembly between GFP1-9 and GFP10-11 engineered to the first loop of ubiquitin. (**B**) SDS PAGE gel analysis of GFP1-9, Ub/GFP10-11, and the complex between GFP1-9 and Ub/GFP10-11. (**C,D**) Stoichiometric binding between GFP1-9 and Ub/GFP10-11. At non-saturating concentrations, the fluorescence intensity increased linearly with the increase of Ub/GFP10-11 protein concentration. (**E,F**) Kinetics for the assembly between 1000 nM GFP1-9 and 1000 nM Ub/GFP10-11. Fluorescence signal intensity plateaus in less than 5 min.

We first evaluated the assembly between GFP1-9 and GFP10-11 in solution. We engineered GFP10-11, along with flanking tetra-glycine linkers at each side of the β-strands with a total molecular weight of ~4 kDa, to the first loop of ubiquitin, between residues Leu8 and Thr9, and we purified the resulting ubiquitin/GFP10-11 protein. We mixed GFP1-9 with ubiquitin/GFP10-11 protein, and the two GFP fragments readily assembled (Fig. 2A), yielding a new band on the SDS PAGE gel (Fig. 2B). At sub-stoichiometric ratios, the GFP fluorescence scaled linearly with the amount of GFP10-11 added (Fig. 2C,D), which indicated that the binding affinity between the two GFP fragments should be sub-nanomolar. Importantly, upon mixing GFP1-9 with ubiquitin/GFP10-11, the intensity of fluorescence increased rapidly and plateaued in less than 5 min (Fig. 2E,F). In contrast, when mixing GFP1-9 with ubiquitin or with HEK293 whole-cell lysate, no GFP fluorescence was observed (data not shown). As such, GFP1-9 specifically and tightly interacts with the GFP10-11 engineered to a protein loop, and the GFP fluorescence can be rapidly turned on upon the assembly of the two fragments.

### Cell surface labeling of GPR17 using the split GFP scheme

**Figure 3:**
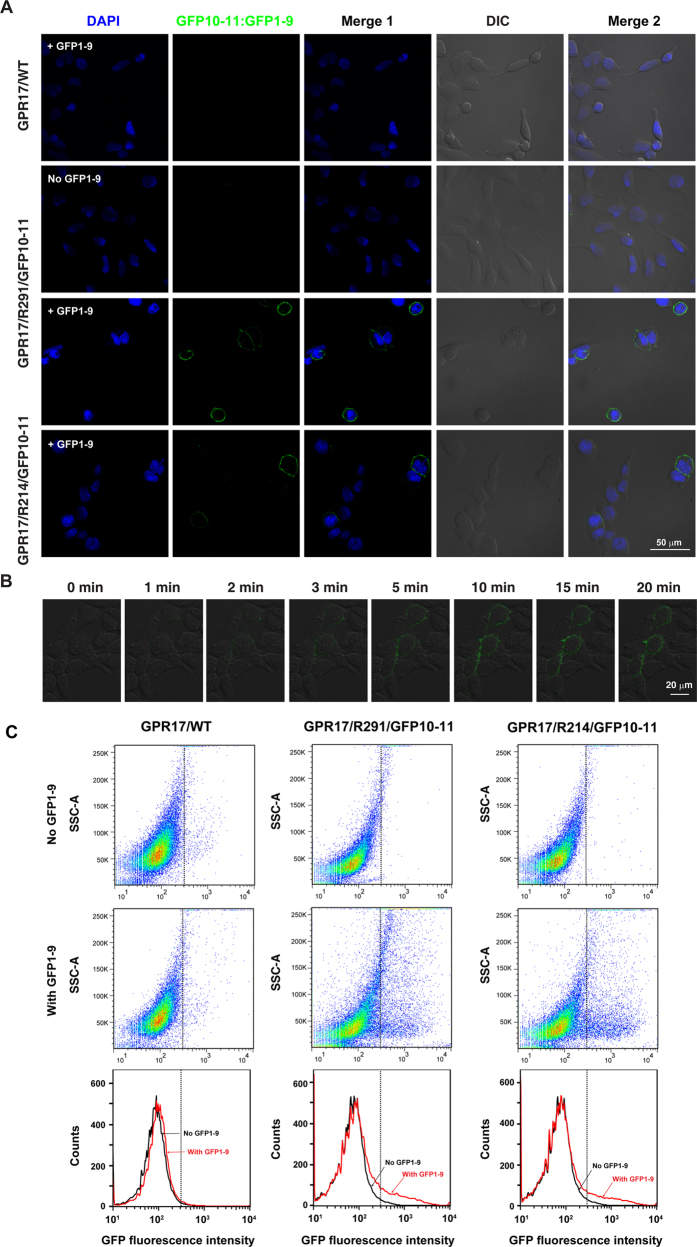
Surface labeling of GPR17 in HEK293 cells. (**A**) Representative images of the cells transfected with wild type GPR17 (GFP17/WT), GPR17/R214/GFP10-11, or GPR17/R291/GFP10-11, in the presence or absence of GFP1-9. (**B**) Kinetics of the assembly between GFP1-9 and GPR17/R291/GFP10-11 in living cells, characterized by the increasing green fluorescence signal at the cell surface. (**C**) Flow cytometry measurements of GPR17. The fluorescence intensity at 488 nm was quantified for cells transfected with GPR17/WT, GPR17/R291/GFP10-11, or GPR17/R214/GFP10-11 in the absence or presence of GFP1-9.

GPR17 is a P2Y-like GPCR that responds to both uracil nucleotides and cysteinyl leukotrienes^17^. We engineered the GFP10-11 tag to the third extracellular loop of GPR17 after residue Arg291 (GPR17/R291/GFP10-11, Fig. 1D) or to the second extracellular loop after residue Arg214 (GPR17/R214/GFP10-11). The cells transfected with GPR17/R214/GFP10-11 or GPR17/R291/GFP10-11 were incubated with GFP1-9 for 20 min. As a control, the cells were also transfected with wild type GPR17. GFP fluorescence was observed for cells transfected with GPR17/R214/GFP10-11 or GPR17/R291/GFP10-11, but not for the cells transfected with wild type GPR17 (Fig. 3A). Significantly, GFP fluorescence was observed only at the surface of transfection-positive cells, with a distribution matching the cell contours in differential interference contrast (DIC) microscopy images (Fig. 3B). In comparison, for cells transfected with a GPR17 engineered with a full-length GFP at the third intracellular loop or a GPR17 engineered with a full-length mCherry at the N-terminus, fluorescence was observed in the entire cell (Fig. S1A–C).

The assembly between GFP1-9 and GPR17/R291/GFP10-11 also occurs rapidly in living cells. Continuous imaging showed that GFP fluorescence appeared in less than one minute after the application of GFP1-9, and continued to intensify until reaching a plateau after about 5 min (Fig. 3B). Of note, during the continuous imaging process, GFP1-9 was added in excess and was not washed off; 20 min after the application of GFP1-9, no fluorescence was observed in the neighboring cells that were transfection negative, and no fluorescence was observed in the glass bottom. Thus the absence of background fluorescence in the neighboring cells testifies the specificity of our split-GFP labeling scheme (Fig. 3B). The fluorescence of the surface-labeled GPR17 could also be assessed using flow cytometry. After the incubation with GFP1-9, increased fluorescence intensity could be quantified for cells that were transfected with GPR17/R214/GFP10-11 or GPR17/R291/GFP10-11, but not for cells transfected with wild type GPR17 (Fig. 3C).

For comparison, we used the sfGFP split into GFP1-10 and GFP11^10^,^15^, with GFP11 genetically incorporated into GPR17 after Arg291 (GPR17/R291/GFP11). We found that GFP1-10 alone has strong fluorescence, and emits ~38% fluorescence as the full-length sfGFP does (Figure S2A). As a result, when administering GFP1-10 to the cell culture medium, background fluorescent dots were observed either around the cells or on the glass cover-slide (Figure S2B). Significantly, no specific fluorescent labeling was observed after the application of GFP1-10 in the cells positively transfected with GPR17/R291/GFP11 (Figure S2B).

The engineering of GFP10-11 to the second or third extracellular loop of GPR17 caused minimal perturbation to the expression and activity of GPR17. Both GPR17/R291/GFP10-11 and GPR17/R214/GFP10-11 positively responded to 100 μM uracil diphosphate (UDP, a specific agonist of GPR17), as characterized by an increase of the intracellular calcium level (Figure S3A,B). As the cells were transiently transfected, they displayed cell-specific responses to UDP with great variations. Considering the variations, the cells transfected with mutant GPR17 had similar responses to UDP as the cells transfected with wild type GPR17 (Figure S3C).

### Internalization of GPR17 and CysLT_2_R visualized with split GFP labeling scheme

**Figure 4:**
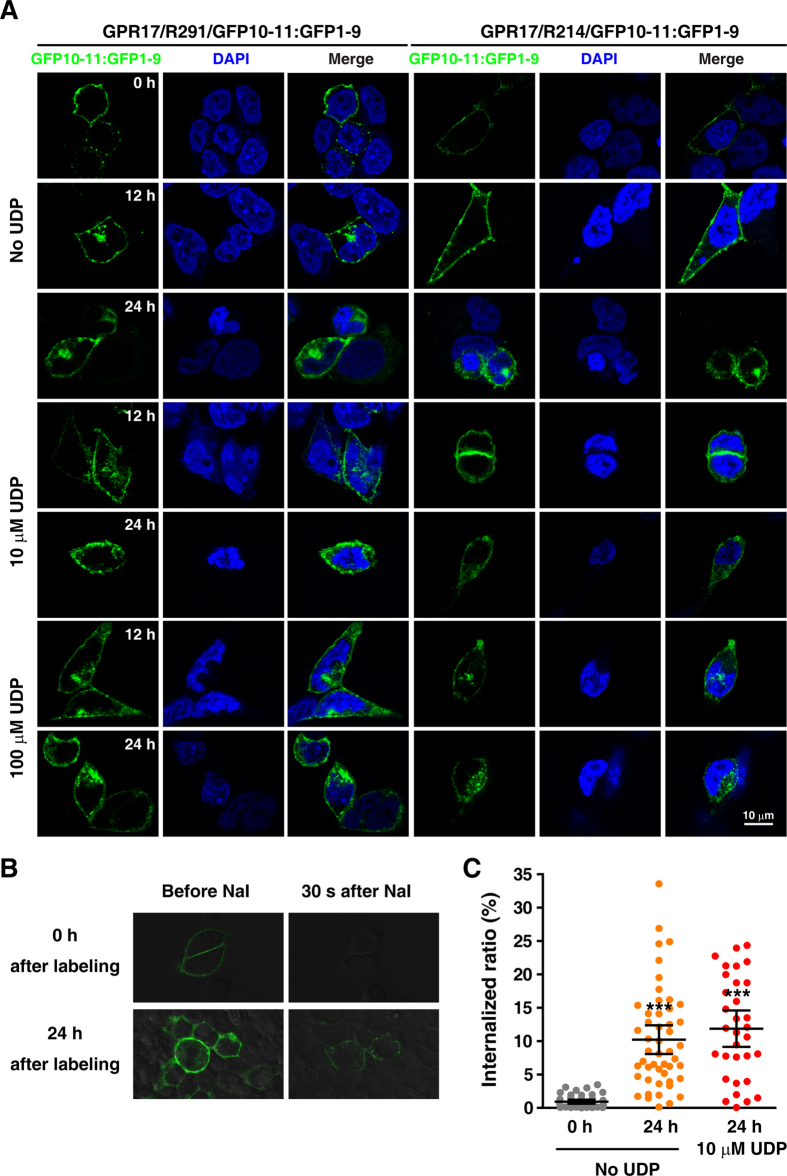
Visualization the internalization of GPR17. (**A**) Representative images of the split GFP labeled HEK293 cells at 0 h, 12 h and 24 h in the absence or presence of UDP, a GPR17 ligand. (**B**) Representative images of the split GFP labeled living cells before and 30 s after the administration of 2 M NaI. The NaI was applied either right after or 24 h after split GFP labeling. (**C**) The statistical analysis of GPR17 internalization for cells with split GFP labeling. ***P < 0.001, compared with 0 h after split GFP labeling with no UDP, one-way ANOVA.

The fact that only GPR17 at the cell surface can be labeled with our split-GFP scheme allowed us to visualize the GPCR internalization processes. After a brief incubation with GFP1-9, green fluorescence became visible for cells positively transfected with GPR17/R214/GFP10-11 or GPR17/R291/GFP10-11. Subsequently, the labeled cells were cultured for 12–24 h in the presence or absence of UDP, diffuse or punctate fluorescence became visible intracellularly, indicative of internalized GPR17 (Fig. 4A).

Iodide ion is a common chemical quencher of fluorescence. We found that the fluorescence of the reassembled split-GFP at the cell surface was completely quenched upon the addition of sodium iodide (NaI). On the other hand, the internalized split GFP labeled GPR17 remained fluorescent, and was not quenched by NaI (Fig. 4B). Thus, by measuring the relative fluorescence intensity before and after the addition of NaI, the internalized GPR17 can be quantitated. We found that even in the absence of UDP, ~10% GPR17 was internalized in 24 h, while the addition of 10 μM UDP did not change the amount of GPR17 internalized (Fig. 3C).

The split-GFP labeling scheme was also demonstrated with another GPCR, cysteinyl leukotriene type-2 receptor (CysLT_2_R). We engineered GFP10-11 tag at two different positions of CysLT_2_R, either at the second loop following residue Gly182 (CysLT_2_/G182/GFP10-11) or at the third loop following residue Lys275 (CysLT_2_/K275/GFP10-11). Specific cell-surface labeling for CysLT_2_R was observed using our split GFP scheme. Upon the addition of 100 nM leukotriene D_4_ (LTD_4_, a specific agonist for CysLT_2_R), internalization of CysLT_2_R could be visualized (Fig. S4).

## Discussion

We have illustrated that a split GFP scheme can be used to label GPCRs at the surface of living cells. Our labeling scheme has these three salient features.

First, the labeling is highly specific, which labels only the genetically tagged GPCRs expressed at cell surface. We further demonstrated that the internalization of GPCRs could be quantitated using a fluorescence quencher. The labeling specificity is owing to the tight affinity between GFP1-9 and GFP10-11 tag, and owing to the fact that the two fragments are non-fluorescent on their own. Once assembled, the noncovalent complex formed between GFP1-9 and GFP10-11 does not dissociate under native conditions, and remains intact during the internalization process of the labeled receptor. With a molecular weight of ~24 kDa, GFP1-9 could not be possibly transported into the cell for direct labeling of intracellular proteins.

Second, the labeling is simple and rapid. GFP fluorescence for GPCR-transfected cells was observed within minutes after the addition of GFP1-9 to cultured medium. In contrast, GFP fluorescence could only be detected hours after the assembly between GFP1-10 and GFP11 fragments in all previous reports^9^,^13^,^15^,^18^. This temporal discrepancy is due to the different methods to prepare GFP fragments. In the previous studies, the fragments were expressed separately. Without the protection of the β-barrel structure in the full-length protein, the GFP fluorophore could not properly mature and fluoresce^19^. As a result, it takes hours for the fluorophore to mature subsequent to the assembly between the two complementary GFP fragments. Boxer and colleagues introduced a proteolytic site between the β-stands 10 and 11, and obtained a mature fluorophore-containing GFP fragment upon cleavage^20^,^21^. However, they simply investigated the photochemical properties of this split-GFP and did not further explore its applications in protein labeling. Here building upon this split-GFP concept and grafting two β-strands of sfGFP to a target protein, we showed that the assembly between GFP1-9 and GFP10-11 could rapidly turn on GFP fluorescence. This feature is essential for monitoring GPCR internalization, as some GPCRs are known to internalize within minutes^3^,^22^.

Third and most importantly, our split GFP labeling scheme allows versatile labeling of a GPCR at multiple sites. An ideal label should cause minimal perturbation to the expression, trafficking, structure and function of a target protein. Previously, sfGFP was split between β-stands 10 and 11, affording GFP1-10 and GFP11^20^,^21^. GFP11, a single β-strand, could be appended to the N-terminus or C-terminus of a target protein, a situation known to perturb the function of some GPCR proteins^8^. The insertion of a stiff β-strand to a protein loop can interfere with the proper folding of a target protein. Alternatively, the target protein may distort the structure of the inserted β-strand, preventing it from interacting with the complementary GFP fragment. Indeed, we showed that tagging GFP11 to the extracellular loop of GPR17 did not lead to specific cell surface labeling. In addition, a GFP1-10 fragment proteolytically derived from the mature sfGFP fluoresces ~40% as the full-length GFP does, and the addition of GFP1-10 to living cell causes large fluorescence background. This explains why GFP1-10 instead of GFP11 was engineered to the target proteins, even though GFP fluorescence could only be observed hours after the reassembly of the split GFP^15^. Here we introduced β-stands 10 and 11 to one of the three GPCR extracellular loops at an optimal insertion site. The GFP10-11 protrudes out with a large degree of flexibility and causes little perturbations to GPCR structure and function. With all these features, we envision that our scheme can be used for surface labeling of other membrane proteins with complex topologies.

## Methods

### Preparation of GFP1-9

A trypsin digestion site with flanking flexible residues was inserted between the 9^th^ and 10^th^ β-strands of sfGFP. The sequence is shown below; the inserted residues are underlined and the cleavage site is denoted with the ▼ symbol.

MGSSHHHHHHSSGLVPGGSHMGGTSSKGEELFTGVVPILVELDGDVNGHKFSVRGEGEGDATIGKLTLKFISTTGKLPVPWPTLVTTLTYGVQAFSRYPDHMKRHDFFKSAMPEGYVQERTISFKDDGKYKTRAVVKFEGDTLVNRIELKGTDFKEDGNILGHKLEYNFNSHNVYITADKQKNGIKANFTVRHNVEDGSVQLADHYQQNTPIGDG**TR**▼**GSGSIEGRHSGSGS**PVLLPDNHYLSTQTVLSKDPNEAGTRGSGSIEGRHSGSGSRDHMVLHEYVNAAGITHGMDELYKGSGGT

The trypsin digestion site was engineered in the loop between β-strands 9 and 10. This modified sfGFP was cloned to the pET-15b vector, and was expressed in BL21 Star (DE3) cells (Stratagene, La Jolla, CA, USA). The cells were induced with 0.5 mM IPTG when the OD_600nm_ value reached 0.8, and were grown for an additional 20 h at 19 °C. The cells were centrifuged at 9000 g for 10 min, resuspended in lysis buffer (300 mM NaCl and 10% glycerol in 50 mM Tris•HCl buffer at pH 8.0), and lysed with a homogenizer. Cell lysate was spun down at 48000 g for 30 min, and the supernatant was collected and loaded on to a Ni:NTA column (GE healthcare, Piscataway, NJ, USA) pre-equilibrated with the lysis buffer. The column was washed with the lysis buffer in the presence of 20 mM imidazole, and the protein was then eluted with 200 mM imidazole. After removing the salt, the protein was further purified on a monoQ column (GE Healthcare), with a gradient from 0 to 200 mM NaCl. The eluent was collected and the buffer was exchanged to trypsin digestion buffer (20 mM Tris, 500 mM NaCl. pH 7.4). 1 U/ml of lyophilized trypsin (from porcine pancreas, Sangon Biotech, China) was used to digest 1 mL of 50 μM sfGFP for 0.5 h. Benzamidine trap (GE Healthcare) and anion exchange columns were used to remove excess trypsin. The digested sfGFP was denatured in 3 M guanidine•HCl at 4 °C for 2 h; GFP1-9 was separated from GFP10-11 on a Superdex75 size exclusion column (GE Healthcare) in denaturing buffer (300 mM NaCl, 3 M guanidine•HCl in 50 mM Tris•HCl buffer at pH 8.0). GFP1-9 was refolded through dialysis against refolding buffer (100 mM NaCl, 10% glycerol in 50 mM Tris•HCl at pH 8.0) at 4 °C overnight. Protein purity and identity were confirmed by SDS-PAGE and by ESI-MS.

### Preparation of GFP10-11 Encoded Ubiquitin (Ub/GFP10-11)

Starting from a pET-11a vector encoding ubiquitin, the cloning of Ub/GFP10-11 was accomplished with three rounds of polymerase chain reaction (PCR). Ultimately, the 10^th^ and 11^th^ GFP β-strands with tetra-glycine linkers on both sides (GGGGGNHYLSTQTVLSKDPNEKRDHMVLHEYVNAAGITGGGG) was engineered between residues Leu8 and Thr9 of human ubiquitin. The plasmid encoding Ub/GFP10-11 was transformed to BL21 Star (DE3) cells. The Ub/GFP10-11 protein was expressed and purified via an SP ion-exchange fast-flow column, a S100 size exclusion column, and a monoS ion exchange column (GE healthcare), in tandem. Protein purity and identity were confirmed by SDS-PAGE and by ESI-MS.

### Characterizing the Assembly of GFP1-9 and Ub/GFP10-11

Proteins including 40 μM GFP1-9, 80 μM Ub/GFP10-11, and a mixture of 40 μM GFP1-9 and 80 μM Ub/GFP10-11, in a total volume of 10 μl were separated by 12% SDS-PAGE without boiling. The gel was stained with 0.1% Coomassie Blue R250. After distaining, the gel was imaged using a Bio-Rad Universal Hood II (Bio-Rad, Hercules, CA, USA).

Fluorescence spectroscopy was performed on a Horiba FluoroMax-4 fluorometer (Japan) in a 600-μL quartz cuvette. The UV absorption and emission spectra was determined for GFP1-9, for the complex between GFP1-9 and Ub/GFP10-11 (1:2 molar ratio), and for the wild type GFP. 20 μM protein concentration was used in the analysis of the UV absorption spectra, while 1 μM protein concentrations was used for the fluorescence emission spectra. After mixing 1 μM GFP1-9 with 2 μM Ub/GFP10-11 protein, fluorescence emission spectra from 460 nm to 600 nm (excited at 450 nm) were recorded at 1-min intervals. The intensity at 507 nm was analyzed and plotted versus time. The binding stoichiometry between GFP1-9 and Ub/GFP10-11 was analyzed by recording fluorescence emission spectra with 1 μM GFP1-9 mixed with different concentrations of Ub/GFP10-11 for 50 min. The intensity at 507 nm was plotted over the concentration of Ub/GFP10-11.

### Construction of GFP10-11 Encoded GPR17

The nucleotide sequence of the human GPR17 gene (Uniprot identifier: Q13304-1) was synthesized (Sangon Biotech) and was cloned to the pcDNA3.1 vector. GFP10-11 with four glycine residues on each side was inserted, to either the 2^nd^ extracellular loop of GPR17 following residue Arg214 (GPR17/R214/GFP10-11), or to the 3^rd^ extracellular loop of GPR17 following residue Arg291 (GPR17/R291/GFP10-11).

### Cell Culture and Transfection

HEK293 cells were purchased from the Institute of Cell Biology of the Chinese Academy of Sciences. Cells were cultured in DMEM supplemented with 10% fetal bovine serum (Sijiqing, China) and 100 U/ml penicillin/streptomycin (Gibco, Grand Island, NY, USA) at 37 °C in a humidified atmosphere containing 95% air and 5% CO_2_. The transfection was performed with lipofectamine-2000 (Invitrogen, Waltham, MA, USA) according to the manual. Briefly, one day before transfection, HEK293 cells were plated in a 6-well plate. When the cells grew to 80–90% confluence, the medium was changed to opt-MEM (Gibco) for transfection. 6 h after transfection, the opt-MEM was changed to DMEM containing serum and penicillin/streptomycin. Cells were passed to 24-well plates with a round glass cover-slide (for imaging), or passed to 35 mm^2^ bottles and cultured for another 1–2 days (for flow cytometry analysis).

### Cell Surface Labeling of GPR17 with Split GFP

2 days after transfection with wild type GPR17, GPR17/R214/GFP10-11, or GPR17/R291/GFP10-11, the cells growing on the cover slides were washed twice with PBS, and incubated with 2 μM GFP1-9 in PBS at 37 °C for 20 min. After the PBS wash, the cells were fixed with freshly prepared 4% paraformaldehyde at 37 °C for 10 min; the cover slides were mounted on glass slides with an anti-fade medium containing DAPI (Invitrogen). Images were captured using an A1 confocal laser-scanning microscope (Nikon, Japan). Simultaneous dual fluorescence acquisitions were performed using the 405 nm and 488 nm lasers to excite DAPI and GFP, respectively. To visualize fluorescence signals in living cells, a cover-slide on which cells were growing was screwed onto a homemade dish. DIC and GFP fluorescence images for the living cells were captured at 0, 1, 2, 3, 5, 10, 15 and 20 min after the addition of 2 μM GFP1-9.

### Monitoring the Internalization of GPR17

2 days after transfection with GPR17/R214/GFP10-11 or GPR17/R291/GFP10-11, the cells growing on cover slides were washed twice with PBS and were incubated with 2 μM GFP1-9 in PBS at 37 °C for 20 min. With excess GFP1-9 washed off, the cells were cultured in DMEM with 10% fetal calf serum and penicillin/streptomycin for another 12 h or 24 h, in the presence or absence of 10 μM or 100 μM UDP (Sigma, St. Louis, MO, USA). The cells were then fixed, mounted, and imaged.

To quantitate the relative ratio of internalized GPR17, living cell GFP fluorescence images were captured before and 30 s after the application of 2 M NaI. The integrated intensity of GFP fluorescence for each labeled cell was measured using ImageJ (1.48V). The internalization ratio of GPR17 was calculated as the integrated GFP fluorescence intensity after the application of NaI divided by the integrated fluorescence intensity before the application of NaI.

### Flow Cytometry Analysis of Surface-Labeled GPR17

2 days after transfection with GPR17/WT, GPR17/R214/GFP10-11, or GPR17/R291/GFP10-11, the cells growing in 35 mm^2^ bottles were washed with PBS and incubated in 2 μM GFP1-9 for 20 min. After removing excess GFP1-9, the cells were digested with trypsin and collected by centrifugation at 3000 g for 5 min. Washed twice with PBS, the cells were fixed with 75% ethanol at 4 °C overnight, and were filtered through 300 nylon net to remove aggregated cells. Fluorescence intensity (488 nm for excitation and 507 nm for emission) of the cells was analyzed using a FACScan flow cytometer (Becton Dickinson, Franklin Lakes, NJ, USA), with 30,000 cells analyzed for each sample.

### Statistical Analysis

Data was presented as mean with 95% confidence interval (CI). Statistically significant differences between experimental groups were determined by one-way ANOVA with Turkey comparison for all pair of columns using Prism software (GraphPad 5.0, San Diego, CA, USA).

## Additional Information

**How to cite this article**: Jiang, W.-X. *et al.* Specific cell surface labeling of GPCRs using split GFP. *Sci. Rep.* **6**, 20568; doi: 10.1038/srep20568 (2016).

## Supplementary Material

Supplementary Information
