# Supplementary material for: Specific cell surface labeling of GPCRs using split GFP
Source: Sci Rep. 2016 Feb 9;6:20568. doi: 10.1038/srep20568 (PMC4746647; doi:10.1038/srep20568)
Supplement: Supplementary Information [file srep20568-s1.pdf]

Supplemental Information for

Specific cell surface labeling of GPCRs using split GFP

Wen-Xue Jiang[2,+], Xu Dong[2,+],Jing Jiang[2], Yu-Hong Yang[2], Ju Yang[2], Yun-Bi Lu[1], San-Hua Fang[3], Ju Yang[2], Er-Qing Wei[1], Chun Tang[2,*] and Wei-Ping Zhang[1,*]

[1]Department of Pharmacology, Zhejiang University School of Medicine, Hangzhou, Zhejiang 310058, China.

[2]CAS Key Laboratory of Magnetic Resonance in Biological Systems, State Key Laboratory of Magnetic Resonance and Atomic Molecular Physics, Wuhan Institute of Physics and Mathematics of the Chinese Academy of Sciences, Wuhan, Hubei Province 430071, China.

[3]Core Facilities of Zhejiang University Institute of Neuroscience, Zhejiang University School of Medicine, Hangzhou, Zhejiang 310058, China

[+]These authors contributed equally to this work.

[*] Correspondence and requests for materials should be addressed to W.P.Z. (email: weiping601@zju.edu.cn) and C.T. (email: tanglab@wipm.ac.cn)

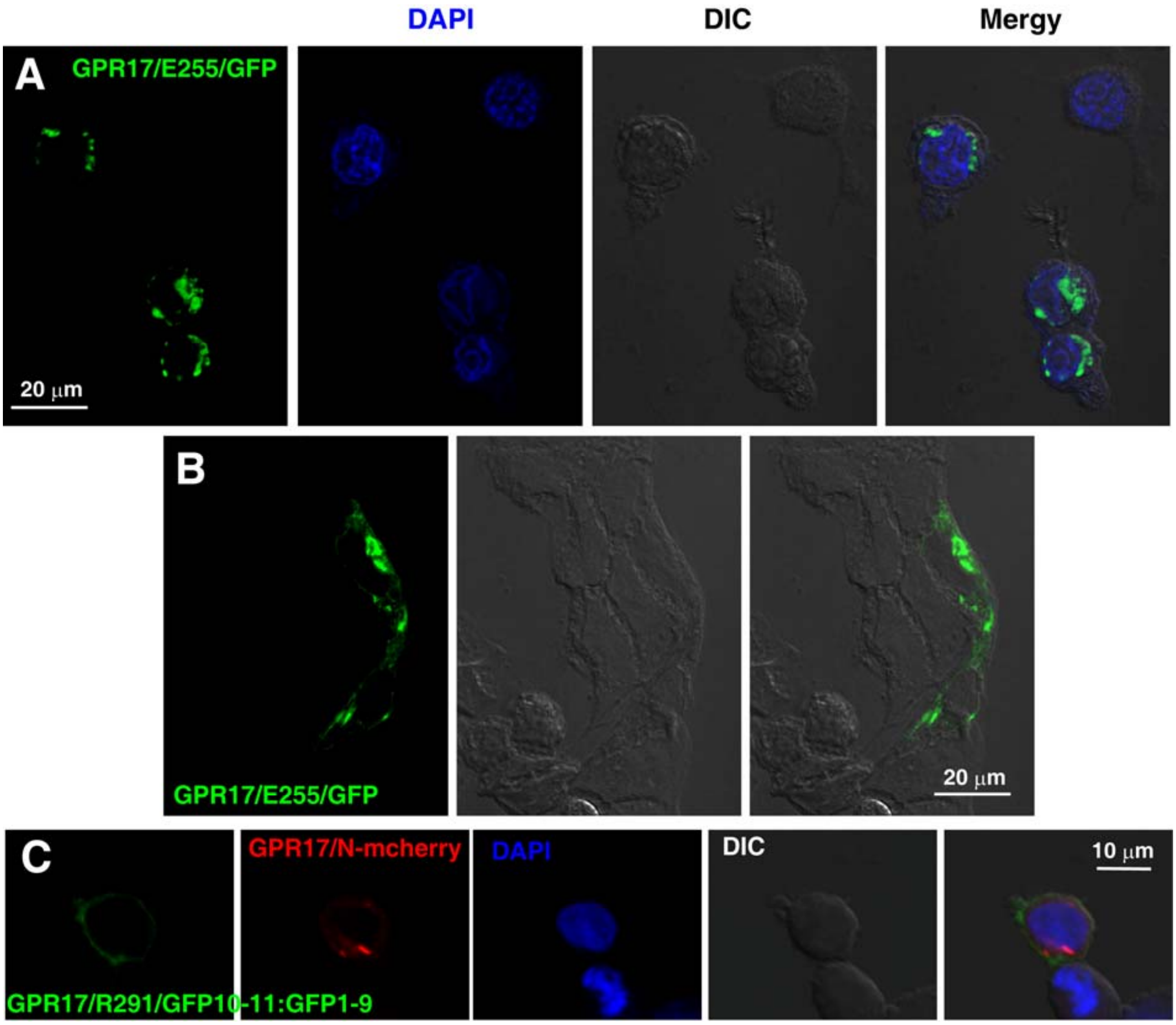

Supplementary Figure S1. Distribution of GFP- and mCherry-fused GPR17 in HEK293 cells. (**A,B**) Representative images of GPR17/GFP positive cells at 48 h after transfection either imaged after fixation (A) or in living cells (B), with the full-length GFP engineered to the 3rd intracellular loop of GPR17 (after residue E255). (**C**) Representative images for GPR17/mCherry transfection positive and split-GFP transfection positive cells at 48 h after transfection. The full-length mCherry was engineered at the N-terminus of GPR17, and the HEK293 cells were doubly transfected with mCherry/GPR17 and GPR17/R291/GFP10-11. Engineering a full-length fluorescent protein affords diffusive fluorescence throughout the cells.

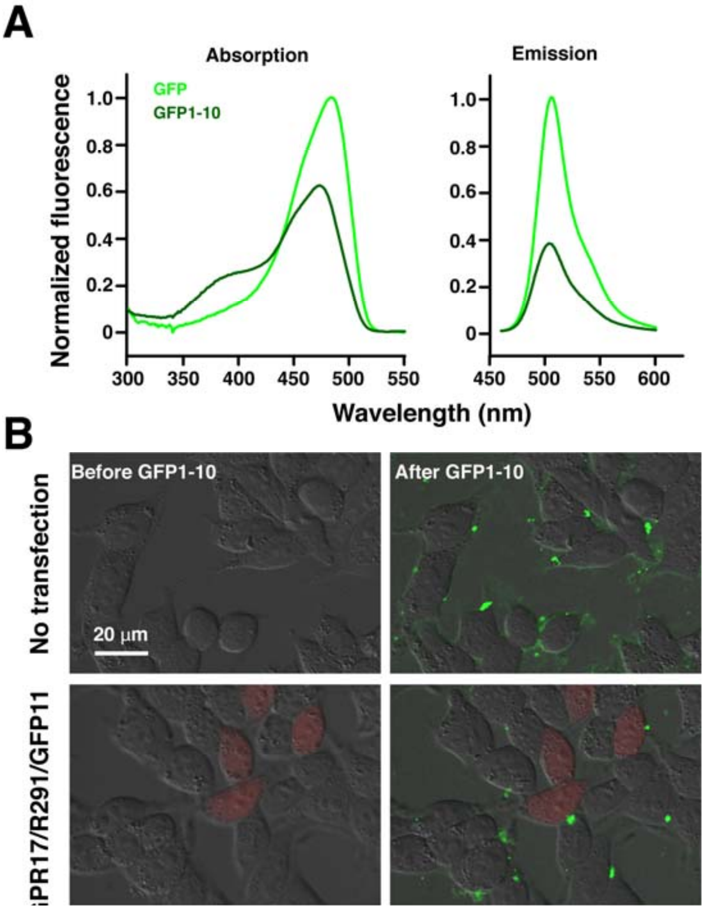

Supplementary Figure S2. The characterization of GFP1-10 and GFP11 split scheme. (**A**) Absorption and fluorescence emission spectra for wild type full length GFP and GFP1-10. GFP1-10 emits ~40% of the fluorescence relative to the full-length GFP. (**B**) Representative images of the cells with no transfection or transfected with GPR17/R291/GFP11 before and after the addition of GFP1-10 (2 μM) to living HEK293 cells. To identify GPR17/R291/GFP11 transfection-positive cells, a co-expression construct was used, in brief, the DNA fragment encoding the mCherry-2A-GPR17/R291/GFP11 gene was cloned into the EcoRI/XhoI sites of pcDNA3.1 (Invitrogen), affording the construct called pcDNA3.1-mCherry-2A-GPR17/R291/GFP11. The mCherry and GPR17 are separated by the sequence of a T2A self-cleaving peptide, yielding the two co-expressed proteins are co-expressed in the same cell at the same amount[1,2]. The construct was verified by restriction digestion and by DNA sequencing. Note that the administration of GFP1-10 does not result in specific labeling of GPR17 at the cell surface but introduces large green fluorescence background.

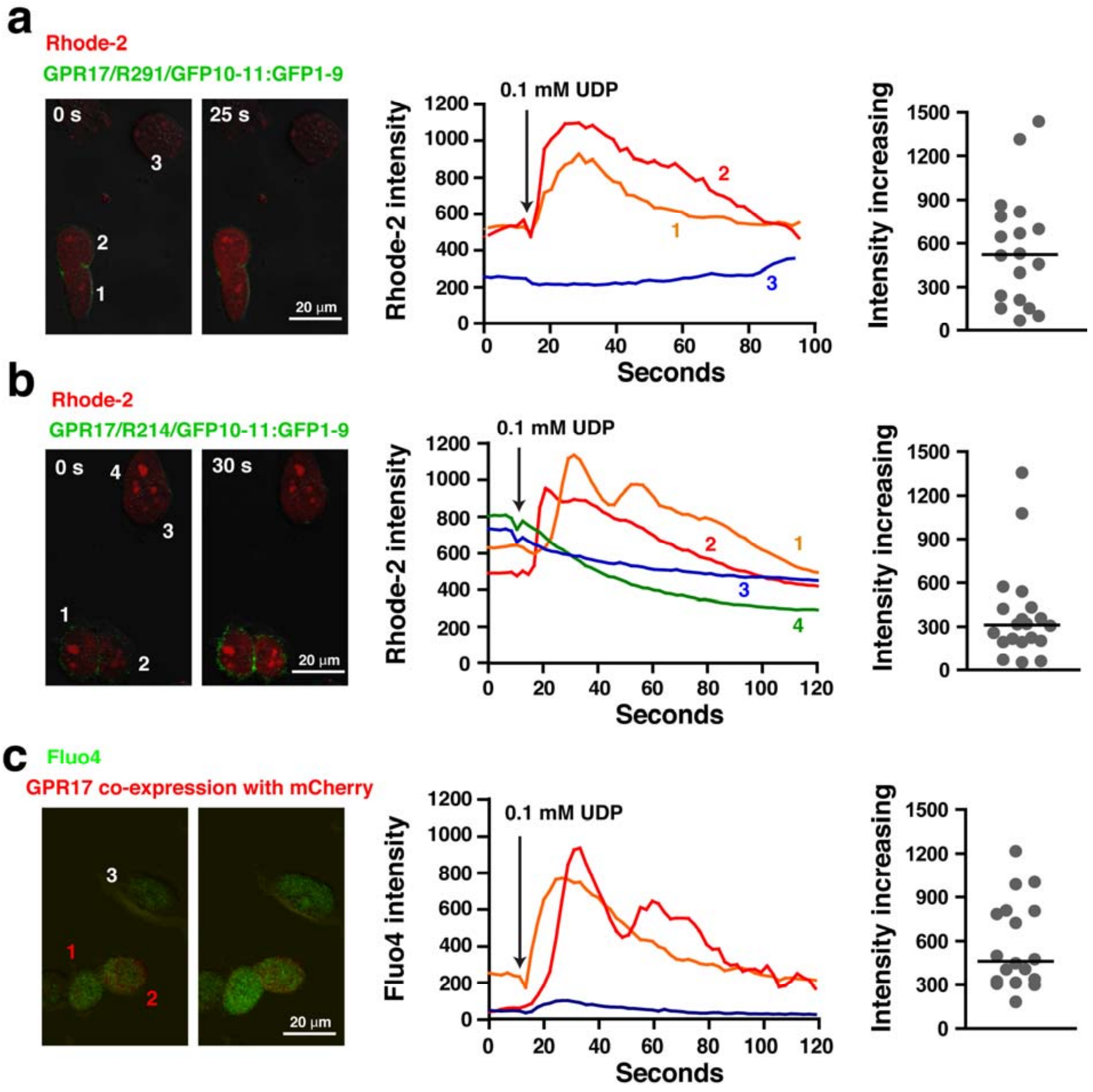

Supplementary Figure S3. Intracellular calcium level in response to UDP. Cells were transfected with (**A**) GPR17/R291/GFP10-11, (**B**) GPR17/R214/GFP10-11, or (**C**) wild type GPR17, and were grown on round glass cover-slides in 24-well plates.

To identify cells positively transfected with wild type GPR17 in (C), the mCherry and GPR17 co-expression plasmid was constructed similar as above. The mCherry and wild type GPR17 are

separated by the sequence of a T2A self-cleaving peptide[1,2], yielding the two co-expressed proteins in the same cells with the same amount. This construct was verified by restriction digestion and by DNA sequencing.

24 h after transfection, the cells transfected with GPR17/R291/GFP10-11 or GPR17/R214/GFP10-11 were incubated with GFP1-9 for 20 min. The cells transfected with GPR17 mutants were incubated with 2.5 mM Rhode-2 (Invitrogen, Waltham, MA, USA), and the cells transfected with wild type GPR17 were incubated with 2.5 mM Fluo4 (Invitrogen, Waltham, MA, USA), both for 45 min. After washing with PBS, live-cell images were taken before the application of 0.1 mM UDP and every second after the application of UDP. Representative images (left), calcium response curves (middle) and the statistical analysis of the calcium responses (right) for cells transfected with (A) GPR17/R291/GFP10-11, (B) GPR17/R214/GFP10-11, and (C) wild type GPR17 are shown.

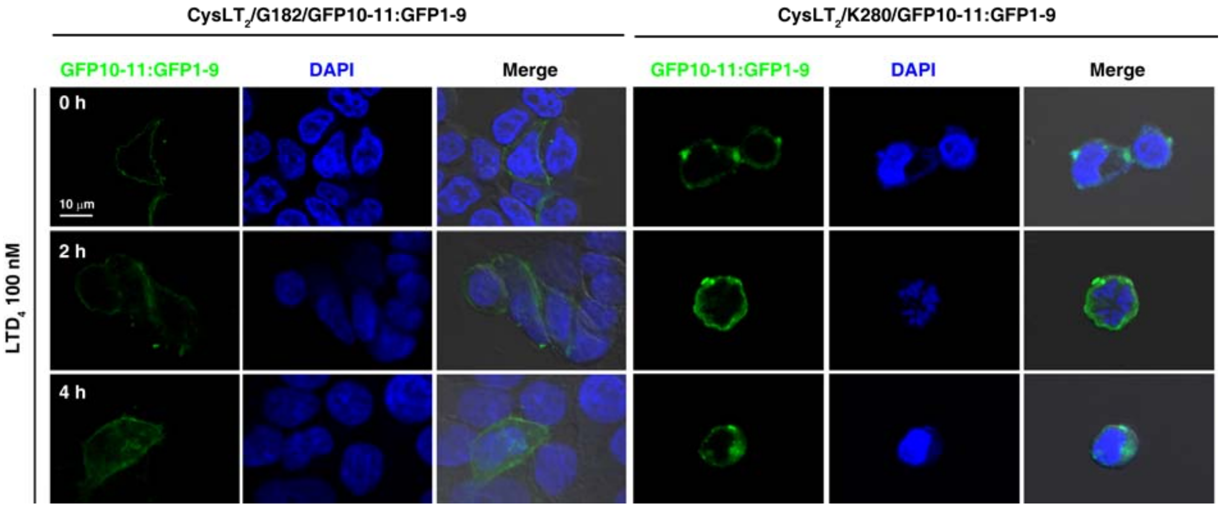

Supplementary Figure S4. Internalization of $CysLT_2R$ in HEK293 cells visualized by surface-labeled by split GFP. HEK293 cells were transfected with $CysLT_2$/G182/GFP10-11 or $CysLT_2$/K280/GFP10-11 and were incubated with GFP1-9. The trafficking of $CysLT_2R$ was monitored at 2 h and 4 h after the addition of LTD_4, a $CysLT_2R$ ligand.

Supplementary references:

1. Kim, J.H., et al., High cleavage efficiency of a 2A peptide derived from porcine teschovirus-1 in human cell lines, zebrafish and mice. *PLoS One*. **6,** e18556 (2011).
2. Szymczak, A.L., et al., Correction of multi-gene deficiency in vivo using a single 'self-cleaving' 2A peptide-based retroviral vector. *Nat. Biotechnol*. **22**, 589-594 (2004).
